# Unrepaired most severe form of hypoplastic left heart syndrome in adulthood: a case report of late diagnosis and long-term follow-up

**DOI:** 10.1093/ehjcr/ytaf621

**Published:** 2025-11-27

**Authors:** José Martín Alanís-Naranjo, Stephanie Teresa Angulo-Cruzado, Edgar García-Cruz, Sergio Alfonso Patrón-Chi, Regina de la Mora-Cervantes

**Affiliations:** Cardiovascular Imaging Department, Instituto Nacional de Cardiología Ignacio Chávez, Juan Badiano 1, Mexico City, 14080, Mexico; Adult Congenital Heart Disease Department, Instituto Nacional de Cardiología Ignacio Chávez, Juan Badiano 1, Mexico City, 14080, Mexico; Adult Congenital Heart Disease Department, Instituto Nacional de Cardiología Ignacio Chávez, Juan Badiano 1, Mexico City, 14080, Mexico; Cardiovascular Imaging Department, Instituto Nacional de Cardiología Ignacio Chávez, Juan Badiano 1, Mexico City, 14080, Mexico; Cardiovascular Imaging Department, Instituto Nacional de Cardiología Ignacio Chávez, Juan Badiano 1, Mexico City, 14080, Mexico

**Keywords:** Adult, Univentricular heart, Hypoplastic left heart syndrome, Mitral atresia, Aortic atresia, Cardiac computed tomography, Case report

## Abstract

**Background:**

Hypoplastic left heart syndrome (HLHS) is a rare, severe congenital heart disease (CHD) characterized by the underdevelopment of the left-sided cardiac structures, typically necessitating neonatal surgical intervention. Survival beyond infancy without surgical palliation is exceptionally uncommon.

**Case summary:**

We report the case of an 17-year-old woman with a late diagnosis of unrepaired HLHS, comprising mitral and aortic atresia, a hypoplastic ascending aorta, and a rudimentary left ventricle. Cardiac imaging provided a comprehensive anatomical assessment. Remarkably, despite the absence of surgical intervention, the patient remained clinically stable over a 10-year follow-up under exclusive medical management.

**Discussion:**

This case illustrates extraordinary natural survival in unrepaired HLHS and emphasizes the importance of specialized CHD centres in long-term management, clinical surveillance, and imaging-based evaluation of complex univentricular physiology.

Learning points

- Survival into adulthood with unrepaired hypoplastic left heart syndrome (HLHS) is exceptionally rare and reflects an atypical yet stable circulatory physiology.
- Advanced cardiac imaging is pivotal for characterizing the anatomical complexity of unrepaired HLHS and guiding clinical management.

## Introduction

The term ‘univentricular heart’ refers to congenital malformations characterised by the absence or severe hypoplasia of one ventricle, precluding biventricular repair. Included entities are tricuspid atresia, hypoplastic right heart syndrome, hypoplastic left heart syndrome (HLHS), double-inlet ventricles, extreme forms of unbalanced complete atrioventricular septal defects, and single ventricles of indeterminate morphology.^1^

Hypoplastic left heart syndrome constitutes the most common form of univentricular heart and one of the most complex and severe congenital cardiac anomalies. Accounting for 2%–3% of all congenital heart disease (CHD) cases, it represents the leading cause of neonatal cardiac mortality, contributing to 25%–40% of fatal outcomes in this population.^2,3^

The syndrome encompasses a spectrum of malformations defined by normally related great arteries, severe obstruction of left ventricular inflow or outflow, and marked underdevelopment of left-sided cardiac structures, including the mitral valve, left ventricle (LV), aortic valve, ascending aorta, and aortic arch, resulting in univentricular circulation.^2,4^

Based on the severity of left ventricular inflow and outflow obstruction, three morphologic subtypes of HLHS are recognised: mitral stenosis with aortic stenosis, mitral stenosis with aortic atresia (AA), and mitral atresia (MA) with AA. All subtypes share the common feature of an underdeveloped LV incapable of supporting systemic circulation.^2^

Foetal survival in HLHS depends on ductus arteriosus patency, which permits the right ventricular output to sustain systemic circulation through the descending aorta.^2^ Without treatment, patients typically die within the first few days or weeks of life; survival beyond the first month remains uncommon, and reports of unrepaired individuals reaching adulthood are exceedingly rare.^5^

We present a case report describing the comprehensive assessment and 10-year follow-up of an adult patient with a late diagnosis of HLHS, characterized by MA, AA, and a hypoplastic ascending aorta.

## Summary figure

**Figure:**
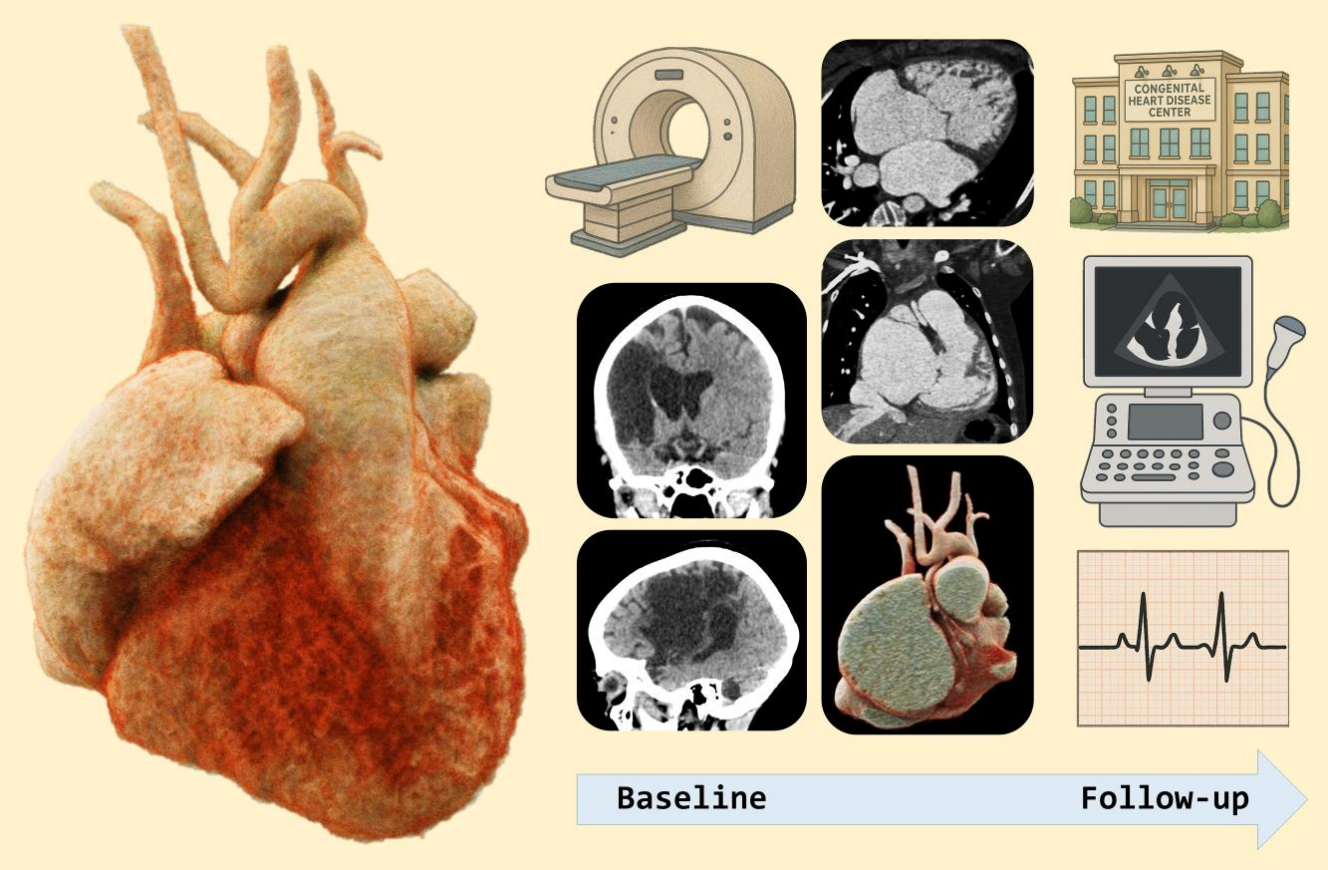


## Case report

A 17-year-old woman was admitted for comprehensive evaluation of suspected CHD due to lifelong cyanosis and a cardiac murmur detected in childhood. Medical history included an ischaemic stroke at age 5, with no subsequent motor, sensory, or cognitive impairments, and no advanced specialist subsequent evaluation to date.

At admission, the oxygen saturation on room air was 85%, while the remaining vital signs were within normal limits, with a heart rate of 60 b.p.m., respiratory rate of 13 b.p.m., and blood pressure of 110/70 mmHg. Physical examination revealed peripheral cyanosis, pulmonary crackles, digital clubbing, along with a continuous Grade 4 murmur with a machine-like quality, most prominent in the left infraclavicular area.

Functional capacity corresponded to NYHA Class II, as the patient was able to perform routine household activities, including cooking, washing, and sweeping, and could climb one to two flights of stairs at a moderate pace without experiencing dyspnoea, while both the patient and family members denied the presence of oedema or any other manifestations of heart failure (HF). Baseline blood tests were unremarkable except for an elevated NT-proBNP level (*Table 1*).

**Table 1:** Baseline and follow-up laboratory parameters

Parameter	Units	Reference range	Baseline (17 years old)	Year 1	Year 3	Year 5	Year 7	Year 8	Year 9	Year 10
Renal function										
Creatinine	mg/dL	0.5–0.9	0.67	0.71	0.59	0.6	0.63	0.69	0.73	0.63
Hepatic function										
AST	U/L	10–35	26.5	28.2	NR	NR	NR	25.5	NR	23
ALT	U/L	10–35	17.9	22.8	NR	NR	NR	20.5	NR	12.9
Total bilirubin	mg/dL	0–0.9	0.55	NR	NR	NR	NR	0.88	NR	0.49
Direct bilirubin	mg/dL	0–0.2	0.15	NR	NR	NR	NR	0.2	NR	0.17
Albumin	g/dL	3.9–4.9	4.4	NR	NR	NR	NR	4.9	NR	4.7
Metabolic										
Glucose	mg/dL	74–106	81	88	82	80	83	84	NR	79
Haematology										
Haemoglobin	g/dL	11.7–16.3	14.8	16	15.9	15.9	15.5	16	15.6	15.6
Haematocrit	%	35.4–49.4	45.2	48.4	47.2	48.2	46.8	49	47.2	44.9
RBC count	10⁶/µL	3.8–5.4	4.85	5.01	5.16	5.06	4.89	5.2	4.97	4.86
WBC count	10³/µL	3.5–10.3	4.9	5	8.4	4.88	6.39	5.06	5	5.42
Platelets	10³/µL	167–431	201	194	221	231	232	228	228	275
Iron studies										
Serum iron	µg/dL	33–193	NR	120	NR	NR	83	NR	127	94
Ferritin	ng/mL	13–150	NR	130	NR	NR	106	NR	128	188
Cardiac biomarker										
NT-proBNP	pg/mL	<125	6657		NR	NR	NR	4319	NR	2305

ALT, alanine aminotransferase; AST, aspartate aminotransferase; NR, not reported, NT-proBNP, N-terminal pro-B-type natriuretic peptide; RBC, red blood cell; WBC, white blood cell.

**Figure 1:**
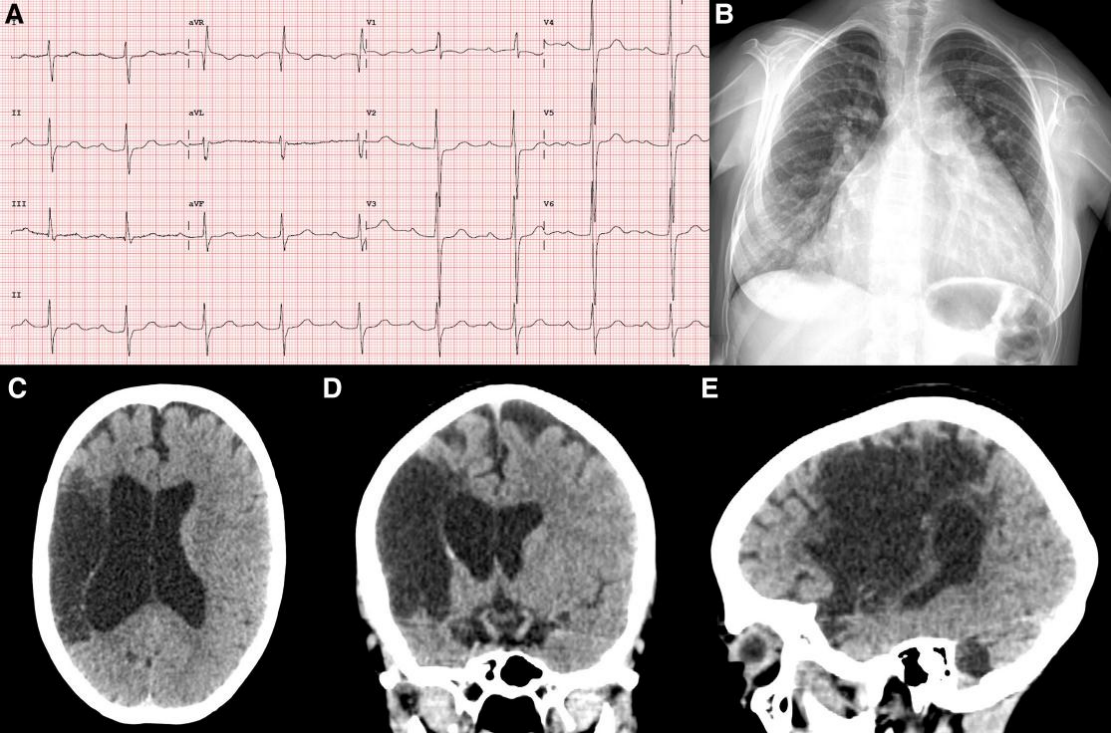
Initial evaluation. (*A*) Electrocardiogram demonstrating first-degree atrioventricular block and right ventricular hypertrophy; (*B*) chest X-ray showing pulmonary congestion and cardiomegaly secondary to right-sided chamber enlargement; (*C–E*) cranial computed tomography demonstrating ischaemic changes in the territory of the middle cerebral artery: (*C*) axial view, (*D*) coronal view, and (*E*) sagittal view.

**Figure 2:**
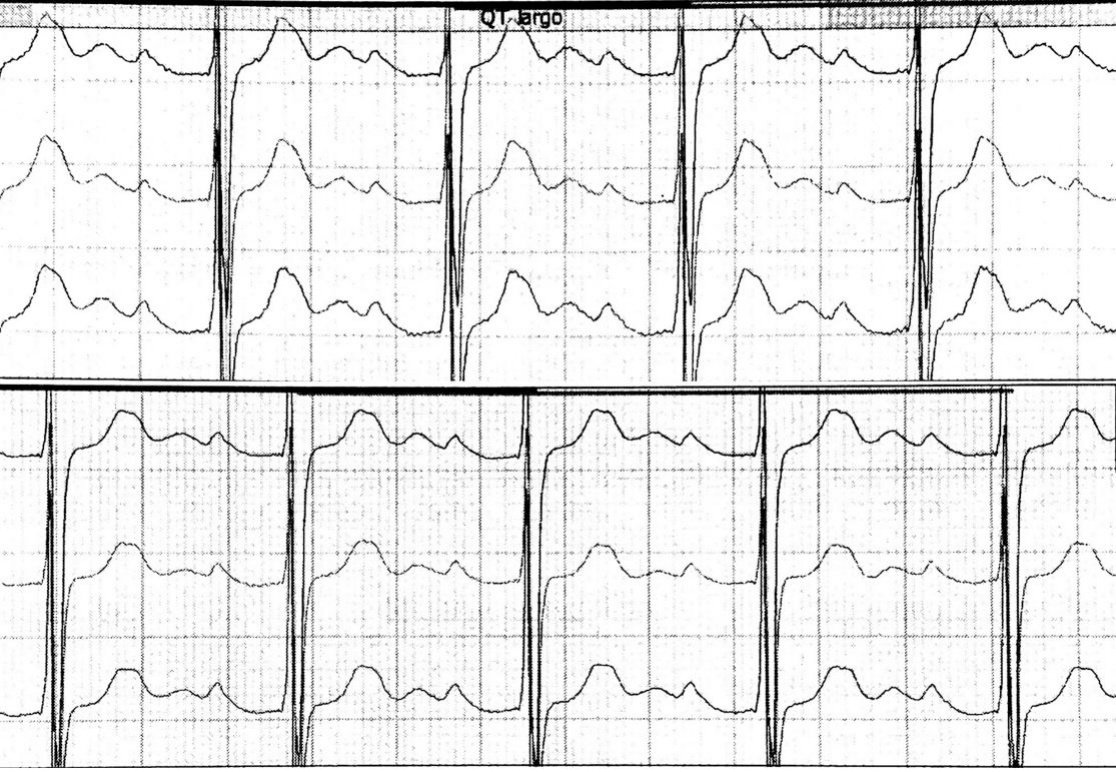
Twenty-four-hour Holter monitor. Atrial flutter with a peak heart rate of 82 b.p.m., a minimum of 40 b.p.m., and an average of 52 b.p.m.

Electrocardiogram showed first-degree atrioventricular block and right ventricle (RV) hypertrophy (*Figure 1A*), while chest X-ray revealed pulmonary congestion and cardiomegaly secondary to right-sided chamber enlargement (*Figure 1B*). Cranial computed tomography demonstrated ischaemic changes in the middle cerebral artery (*Figure 1C–E*). A 24 h Holter study recorded atrial flutter predominant during sleep, with a peak heart rate of 82 b.p.m. (*Figure 2*).

**Figure 3:**
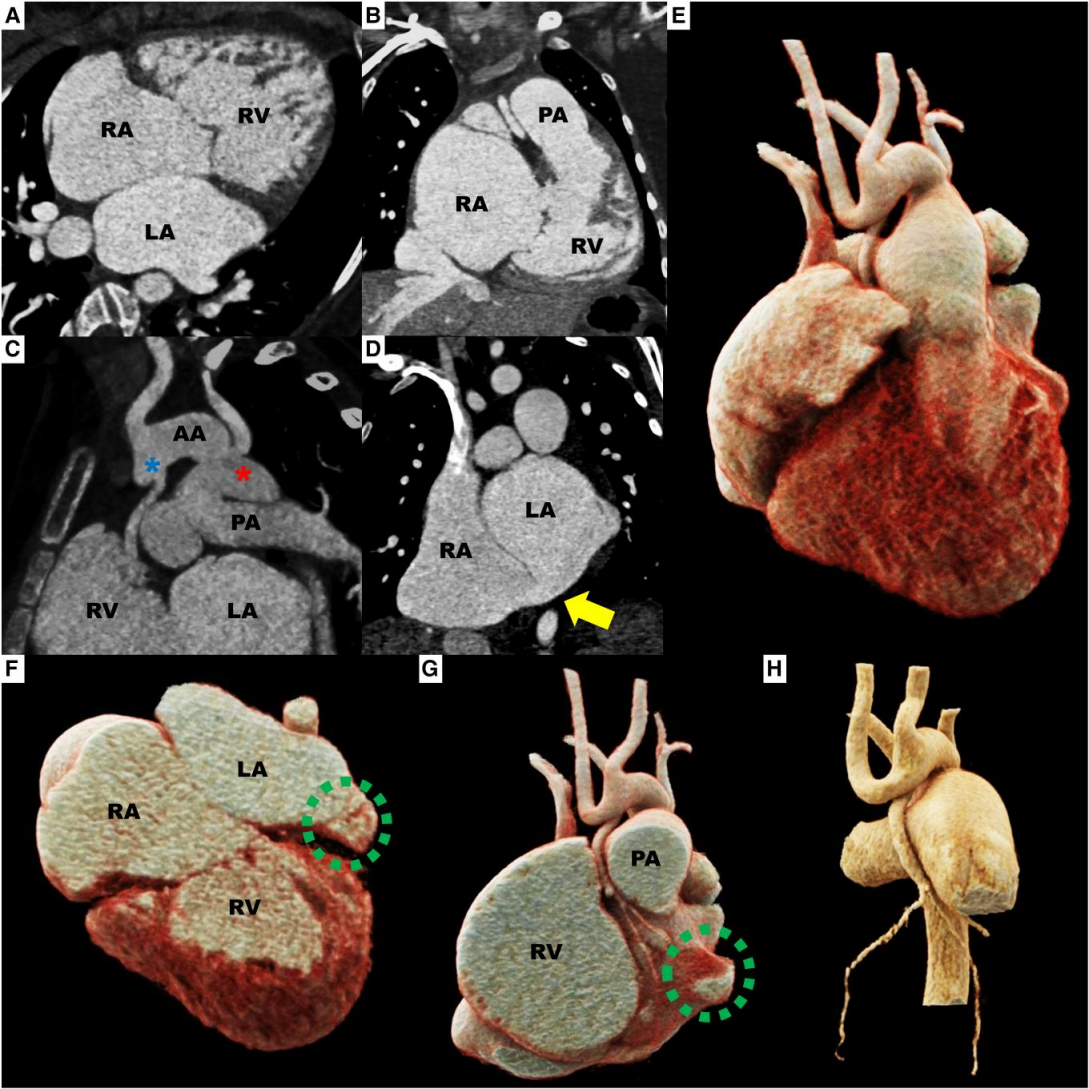
Cardiac computed tomography. Usual atrial arrangement, concordant atrioventricular connections with an imperforate left atrioventricular junction due to mitral atresia, a rudimentary morphologic left ventricle positioned posteriorly and to the left (green dotted line), and ventriculoarterial connections of the single-outlet type from the morphologic right ventricle secondary to AA, sinus venosus atrial septal defect measuring 9 mm × 12 mm (yellow arrow), coronary arteries originating bilaterally from a hypoplastic ascending aorta (blue asterisk), and a prominent patent ductus arteriosus measuring 17 mm × 20 mm at the pulmonary end, 18 mm × 18 mm at the aortic end, and 5 mm in length (red asterisk). (*A*) Axial MPR view; (*B*) coronal MPR view; (*C*) oblique MIP view; (*D*) coronal MPR view; (*E*) cinematic VRT 3D reconstruction in anterior view; (*F*) slice-based cinematic VRT 3D reconstruction in axial view; (*G*) slice-based cinematic VRT 3D reconstruction in coronal view; (*H*) cinematic VRT 3D reconstruction of coronary anatomy. 3D, three-dimensional; AA, ascending aorta; LA, left atrium; MIP, maximum intensity projection; MPR, multiplanar reconstruction; PA, pulmonary artery; RA, right atrium; RV, right ventricle; VRT, volume rendering technique.

**Figure 4:**
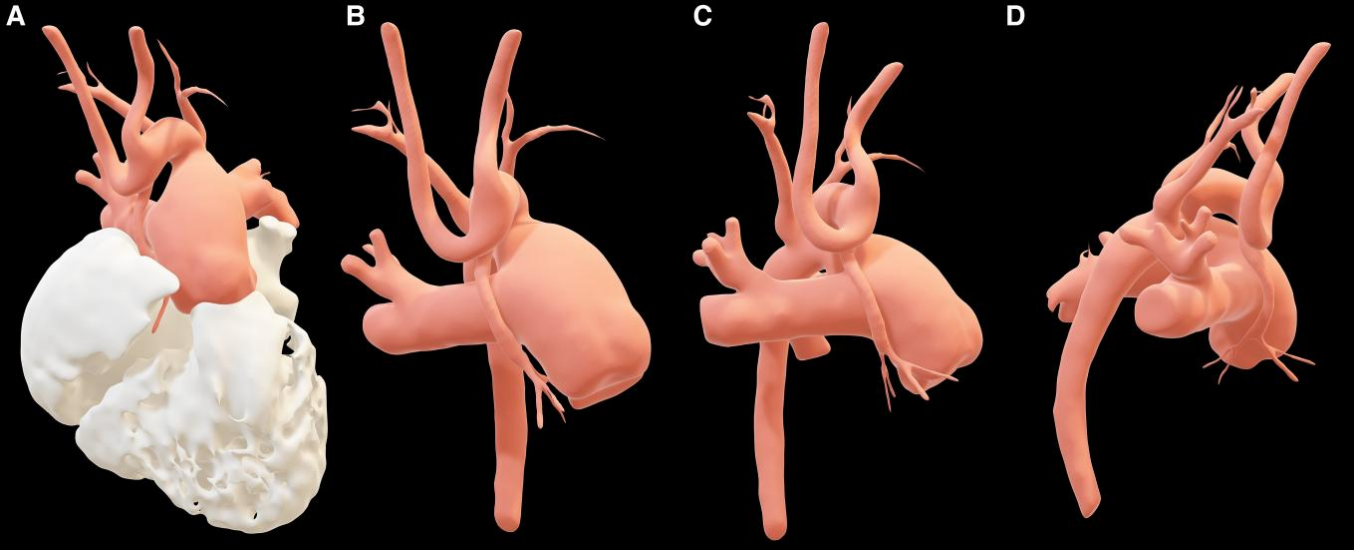
Hypoplastic left heart syndrome. Cinematic VRT 3D reconstruction showing aortic root and great vessels visualised with cardiac chambers (*A*) and in isolated views; (*B*) anterior view, (*C*) left lateral view, and (*D*) posterior view. 3D, three-dimensional; VRT, volume rendering technique. Courtesy of Sergio Alfonso Patrón-Chi, Instituto Nacional de Cardiología Ignacio Chávez, Mexico City, Mexico. Contact: drsergiopatron@gmail.com.

The echocardiogram did not fully delineate the ascending aorta, yet revealed mitral and AA, biatrial enlargement, a sinus venosus atrial septal defect (ASD) with bidirectional shunt, dilatation of the main pulmonary trunk and its branches, a patent ductus arteriosus with right-to-left flow, and retrograde filling of the aortic arch. Cardiac CT confirmed the same findings and further demonstrated a rudimentary LV situated posteriorly, with coronary arteries arising separately from both sides of a hypoplastic ascending aorta (*Figures 3* and *4*).

A Naughton treadmill test workload evaluated the functional class, with the resting ECG at the start showing sinus rhythm at 55 b.p.m. and no arrhythmias; the test continued for 9.5 min, ending due to lower limb fatigue at an estimated workload of 5.4 metabolic equivalents (METs), with no arrhythmias observed and the patient remaining asymptomatic throughout exercise and recovery.

Considering the complex cardiac anatomy and physiology, the long-standing clinical stability, and the socioeconomic circumstances of the patient and her family that could pose challenges to maintaining adherence to the long-term immunosuppressive therapy required for heart transplantation, the Heart Team decided to continue with medical management. The patient was discharged in asymptomatic condition on a treatment regimen consisting of acenocoumarin 7 mg per week, furosemide 20 mg daily, and bisoprolol 2.5 mg daily, with surveillance in our adult CHD department.

During long-term ongoing care, the patient developed progressive tricuspid valve disease without evidence of RV systolic dysfunction (*Table 2*). Medical therapy was adjusted to include spironolactone 25 mg daily and dapagliflozin 10 mg daily, with no further hospitalizations or recurrence of atrial flutter on Holter monitoring. At 10-year ongoing monitoring, clinical status remained stable, with preserved functional capacity corresponding to NYHA Class II and continued engagement in usual daily activities. Laboratory parameters, summarized in *Table 1*, showed a progressive decline in NT-proBNP levels and a stable hepatic and renal function within normal limits.

**Table 2:** Echocardiographic follow-up evaluation

	17 years old	23 years old	25 years old	27 years old
Echocardiogram				
RV FAC, %	NR	35	36	35
TAPSE, mm	20	21	25	25
*S*′, cm/s	12	11	12	14
Indexed right atrial volume, mL/m^2^	153	179	187	NR
Indexed left atrial volume, mL/m^2^	52	56	62	NR
Tricuspid valve disease	Mild regurgitation	Moderate regurgitation	Moderate regurgitation	Moderate regurgitation
Pulmonary valve disease	None	Mild regurgitation	Mild regurgitation	Mild regurgitation

LVEF, left ventricular ejection fraction; NR, not reported; RV FAC, right ventricular fractional area change; S’, tricuspid annular peak systolic velocity; TAPSE, tricuspid annular plane systolic excursion.

## Discussion

Most children with CHD, including complex lesions, survive in adulthood due to advances in congenital cardiology and surgery.^3^ Systemic RV physiology, as in HLHS, is the most common univentricular heart disease, with a prevalence of 2–3 per 10 000 live births.^4^

Hypoplastic left heart syndrome accounts for 25%–40% of neonatal cardiac deaths without intervention; surgical palliation and transplantation have improved survival by nearly 40% at 5, 10, and 15 years.^4^ The aetiology remains uncertain, although 5%–10% of cases are associated with genetic syndromes, most commonly Turner syndrome, which increases mortality.^2^

The RV, functioning as the systemic ventricle, is structurally and physiologically disadvantaged due to reliance on longitudinal shortening, high load sensitivity, and limited adaptive capacity compared with the LV.^4^ Univentricular circulation must manage systemic and pulmonary venous return, causing chronic volume overload, early ventricular dysfunction, progressive atrioventricular valve regurgitation, reduced exercise tolerance, and eventual HF with cyanosis. Rare cases with balanced haemodynamics may reach adulthood without intervention.^1^

Unoperated univentricular hearts may coexist with septal defects, valve malformations, aortic arch abnormalities, pulmonary artery anomalies, discordant connections, anomalous venous return, or heterotaxy syndromes.^1^ In adults with unoperated HLHS, survival depends on pulmonary venous return entering the right atrium via an ASD, passing to the pulmonary circulation, and reaching the systemic circulation via the ductus arteriosus.^5^

The combination of mitral and AA in the reported patient resulted in long-term survival through an exceptionally well-balanced interaction between the systemic and pulmonary circulations, where a bidirectional interatrial communication allowed continuous decompression of the left atrium and unobstructed pulmonary venous drainage, preventing pulmonary congestion, while a patent ductus arteriosus with right-to-left flow from the pulmonary artery to the aorta maintained systemic and coronary circulation despite the absence of left ventricular outflow; preserved univentricular systolic function ensured adequate cardiac output, and the absence of pulmonary overcirculation or venous obstruction reduced ventricular workload and supported oxygen delivery. Collectively, these compensatory adaptations established a remarkably stable haemodynamic state capable of sustaining life far beyond the expected limits for such anatomy.

Presentation includes cyanosis, with oxygen saturation between 75% and 85% since birth.^1,5^ Clinical findings may involve digital clubbing, precordial heave, scoliosis, and arrhythmias. Complications include HF, stroke, brain abscess, thromboembolism, and infective endocarditis.^1^ Comprehensive evaluation using multimodal imaging and invasive studies is essential to guide management.^1^ Transthoracic echocardiography (TTE), cardiac magnetic resonance (CMR), and cardiac computed tomography (CCT) are principal modalities for anatomical and functional assessment, while cardiac catheterisation is indispensable for haemodynamic evaluation and mandatory before Fontan surgery.^1,6^

In stable patients, surgical risk must be weighed against benefit. Fontan completion is reserved for highly selected cases; bidirectional Glenn or systemic-to-pulmonary shunts may improve pulmonary flow. Heart or heart–lung transplantation should be considered in advanced disease without conventional options, although prior surgeries and multisystem involvement increase complexity.^1^

Since staged surgical strategies culminating in Fontan circulation were introduced four decades ago, HLHS survival has improved, with many patients reaching adulthood.^2^ Nevertheless, HLHS carries higher long-term morbidity and greater risk of arrhythmias, thromboembolism, transplantation, and death compared with other systemic RV physiologies.^2,3^

Heart failure represents the leading cause of death in patients with HLHS, driven by factors such as neo-aortic arch obstruction, valvular insufficiency, restrictive atrial septum, arrhythmias, and circuit obstructions;^3,4^ no proven Class I, Level A therapies exist for HF in CHD, making HLHS management uniquely challenging.^4^

Heart transplantation is the only life-prolonging treatment for end-stage HF but carries the highest early and late mortality among single-ventricle and biventricular CHD populations.^2,7^ Multi-organ failure, including hepatic involvement, is common, requiring close monitoring and early pre-transplant evaluation to determine optimal referral timing.^2^

Increasing adult survival in HLHS demands long-term strategies addressing safe physical activity, infective endocarditis prophylaxis, and early counselling on contraception and pregnancy, which is contraindicated in severe cyanosis, poor ventricular function, or pulmonary vascular disease.^1,2^ Surveillance should include clinical examination, oxygen saturation measurement, and laboratory testing for haematologic indices, iron status, and liver and kidney function.^1^

## Conclusion

Adult survival in unoperated HLHS represents an exceptional finding, underscoring the value of long-term specialised follow-up and cardiac imaging assessment in expert CHD centres.

## Lead author biography


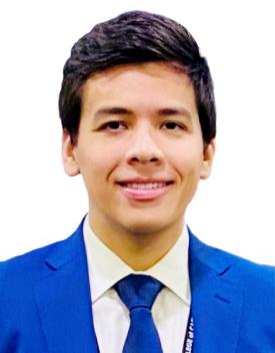


Martín Alanís-Naranjo received his medical degree from the Michoacán University of San Nicolás de Hidalgo in Morelia, Mexico, and later pursued medical specialisation in Internal Medicine at the National Autonomous University of Mexico. He subsequently undertook specialist training in Cardiology at the Hospital Regional 1° de Octubre ISSSTE in Mexico City and is currently serving as a Fellow in Cardiovascular Imaging at the Instituto Nacional de Cardiología Ignacio Chávez in Mexico City.

## Supplementary Material

ytaf621_Supplementary_Data

## Data Availability

The data underlying this article are available within the article and its supplementary materials.

## Supplementary material

**Consent:** The authors confirm that written consent for submission and publication of this case report including images and associated text has been obtained from the patient in accordance with COPE guidelines.

**Funding:** None declared
