# Supplementary material for: Unrepaired most severe form of hypoplastic left heart syndrome in adulthood: a case report of late diagnosis and long-term follow-up
Source: Eur Heart J Case Rep. 2025 Nov 27;9(12):ytaf621. doi: 10.1093/ehjcr/ytaf621 (PMC12723480; doi:10.1093/ehjcr/ytaf621)
Supplement: ytaf621_Supplementary_Data [file ytaf621_supplementary_data.zip › Supplementary Files.docx]

**Supplementary Files**

**Video S1:** Hypoplastic Left Heart Syndrome: Cinematic VRT 3D Reconstruction.
